# Loss of CD11b Exacerbates Murine Complement-Mediated Tubulointerstitial Nephritis

**DOI:** 10.1371/journal.pone.0092051

**Published:** 2014-03-14

**Authors:** Lee Daniel Chaves, Lihua Bao, Ying Wang, Anthony Chang, Mark Haas, Richard John Quigg

**Affiliations:** 1 Division of Nephrology, Department of Medicine, University at Buffalo School of Medicine and Biomedical Sciences, Buffalo, New York, United States of America; 2 Department of Medicine, The University of Chicago, Chicago, Illinois, United States of America; 3 Department of Pathology, The University of Chicago, Chicago, Illinois, United States of America; 4 Department of Pathology and Laboratory Medicine, Cedars-Sinai Medical Center, Los Angeles, California, United States of America; Colorado State University, College of Veterinary Medicine and Biomedical Sciences, United States of America

## Abstract

Acute complement activation occurs in the tubulointerstitium (TI) of kidneys transplanted from Crry^−/−^C3^−/−^ mice into complement-sufficient wildtype mice, followed by marked inflammatory cell infiltration, tubular damage and interstitial fibrosis. We postulated iC3b-CD11b interactions were critical in this TI nephritis model. We transplanted Crry^−/−^C3^−/−^ mouse kidneys into CD11b^−/−^ and wildtype C57BL/6 mice. Surprisingly, there was greater inflammation in Crry^−/−^C3^−/−^ kidneys in CD11b^−/−^ recipients compared to those in wildtype hosts. Kidneys in CD11b^−/−^ recipients had large numbers of CD11b^−^Ly6C^hi^CCR2^hi^F4/80^+^ cells consistent with inflammatory (M1) macrophages recruited from circulating monocytes of the host CD11b^−/−^ animal. There was also an expanded population of CD11b^+^CD11c^+^Ly6C^−^F4/80^hi^ cells. Since these cells were CD11b^+^, they must have originated from the transplanted kidney; their surface protein expression and appearance within the kidney were consistent with the intrinsic renal mononuclear cellular population. These cells were markedly expanded relative to all relevant controls, including the contralateral donor kidney and Crry^−/−^C3^−/−^ mouse kidneys in CD11b^+/+^ wildtype recipients. Direct evidence for their in situ proliferation was the presence of nuclear Ki67 and PCNA in CD11b^+^F4/80^+^ cells. Thus, in this experimental model in which there is unrestricted C3 activation, CD11b^+^ monocytes limit their own infiltration into the kidney and prevent proliferation of endogenous mononuclear cells. This suggests a role for outside-in iC3b-CD11b signals in limiting intrinsic organ inflammation.

## Introduction

Activation of complement through its three pathways leads to generation of C3 and C5 products. These act on a limited set of cellular receptors. C3a and C5a receptors are rhodopsin-like Class A GTP-binding protein-coupled receptors while those that bind C3b and derivatives are termed complement receptors. The latter include the heterodimeric β_2_ integrins, Itgam (CR3, α_M_β_2_, CD11b/CD18) and Itgax (CR4, α_X_β_2_, CD11c/CD18).

The unique rodent complement regulator, CR1-related gene y (Crry) is a structural and functional homologue to human CR1 [1]. Crry is present in endothelial and epithelial cells of the renal tubulointerstitium (TI) in a distribution comparable to membrane cofactor protein in human beings [2], [3]. The relevance of Crry in the TI was first shown by Nomura, Matsuo et al. in rats using neutralizing antibodies [4]. A series of studies from Thurman et al. have shown that the normal polarization of Crry to the basolateral aspect of mouse tubules is lost in ischemia, which leads to unrestricted alternative pathway activation and acute kidney injury upon reperfusion [5], [6], [7]. This appears to be relevant to acute kidney injury (tubular necrosis) in human beings [8].

To evaluate the effects of acute complement activation, we transplanted kidneys from Crry^−/−^C3^−/−^ mice into complement-sufficient wildtype mice. These Crry^−/−^C3^−/−^ kidneys developed TI nephritis with marked inflammatory cell infiltration, tubular damage and interstitial fibrosis [9]. Crry^−/−^C3^−/−^ kidneys transplanted in C3aR^−/−^ hosts were protected from TI nephritis [10]. Thus, acute C3 activation in Crry^−/−^C3^−/−^ kidneys generates C3aR-dependent TI inflammation. Given the prominent infiltration with CD11b^+^ cells in this model, we reasoned that CD11b-iC3b interactions would be relevant. To examine this, we transplanted Crry^−/−^C3^−/−^ mouse kidneys into CD11b^−/−^ recipients.

## Materials and Methods

### Ethics Statement

All animal experimental procedures were carried out in accordance with the National Institutes of Health Guide for the Care and Use of Laboratory Animals and were approved by the Institutional Animal Care and Use Committees of the Universities at Buffalo and Chicago.

### Antibodies

Antibodies used for immunohistological techniques were fluorescein-conjugated anti-mouse C3 (Cappel Pharmaceuticals, Aurora, OH, USA), rat anti-mouse EGF-like module-containing mucin-like hormone receptor-like 1 (EMR1, F4/80, MCA497GA, AbD Serotec, Oxford, England, UK), rat anti-mouse CD11b (M1/70, BD Biosciences, San Jose, CA, USA), rabbit anti-mouse proliferating cell nuclear antigen (PCNA, Santa Cruz Biotechnology, Santa Cruz, CA, USA), and rabbit anti-Ki67 (RM-9106-s, Thermo Fisher Scientific, Waltham, MA, USA).

Antibodies used for flow cytometry were PE-Cy7-conjugated anti-mouse CD11c, APC-Cy7-conjugated anti-mouse CD19, PerCP5.5-conjugated anti-mouse Ly6C/G (Gr1), BD Horizon V500-conjugated anti-mouse CD11b (BD), phycoerythrin-conjugated anti-mouse CCR2 (R&D Systems Minneapolis, MN, USA), and Alexa fluor 647-conjugated anti-F4/80 (AbD Serotec).

### Mice and the TI Nephritis Model

Crry^+/−^ and C3^+/−^ mice [11], [12] were kindly provided by Dr. Hector Molina (Washington University School of Medicine, St. Louis, MO, USA). CD11b^−/−^ mice originally generated by Mayadas et al. [13] were obtained from Jackson Laboratories. Crry deficiency is embryonic lethal due to unrestricted maternal complement activation, which can be averted in Crry^−/−^C3^−/−^ mice [11]. Thus, C3^+/−^ mice were intercrossed to generate C3^−/−^ mice, which were crossed with Crry^+/−^ mice to produce C3^+/−^Crry^+/−^ mice. These were then intercrossed to generate Crry^−/−^C3^−/−^ animals.

The model of TI nephritis relies upon transplantation of kidneys from Crry^−/−^C3^−/−^ mice into C3-sufficient animals [9], [10]. Kidneys from Crry^−/−^C3^−/−^ mice were transplanted into wildtype or CD11b^−/−^ mice (n = 5 each). Both donor and recipient mice were backcrossed onto normal C57BL/6 mice for at least 10 generations. Mice between 8 to 10 weeks old were used as kidney donors or transplant recipients. To limit any potential variation within the model, the entire cohort of mice for the study was raised contemporaneously. Transplant recipients alternated between wildtype and CD11b^−/−^ mice.

Kidney donors were anesthetized and the donor left kidney was removed with artery, vein and ureter attached, and preserved in cold saline on ice. The recipient was then anesthetized and the left kidney was excised. Renal transplantation was performed with end-to-side anastomoses of the donor renal vein, artery and ureter to the recipient inferior vena cava, aorta and bladder, respectively [14]. Total cold ischemic time ranged between 45 and 60 minutes. The left native kidney was removed at the time of renal transplantation. Blood was taken at the time of transplant and then twice over 7 days. Animals were sacrificed 7 days post-transplantation.

In our past control experiments establishing the general transplantation model, wildtype kidneys were transplanted into wildtype recipients (n = 34), along with removal of the second native kidney 7 days after transplantation. In these animals, blood urea nitrogen concentrations were 26.5±0.8 mg/dl 21 days post-transplantation.

### Measurements from Tissue

Four-µm sections of 4% paraformaldehyde-fixed paraffin-embedded kidney tissue were stained with periodic acid-Schiff and examined by a renal pathologist (MH) in a blinded manner. The severity of TI nephritis was graded from 0 to 4 in 0.5 increments [9].

For immunofluorescence microscopy, 4-µm cryostat sections were fixed in ether-ethanol and stained sequentially with anti-mouse F4/80, rhodamine-conjugated goat anti-rat IgG, and fluorescein-conjugated anti-mouse C3.

For immunohistochemistry, zinc-fixed and paraffin-embedded kidney sections were used. Tissue sections were deparaffinized and rehydrated through xylene and serial dilutions of ethanol to deionized water. Endogenous peroxidases and biotin were blocked with 0.3% H_2_O_2_ and Avidin/Biotin Blocking Kit (Vector Laboratories, Burlingame, CA, USA) followed by 10% normal mouse serum. They were incubated in antigen retrieval buffer (S1699, DAKO, Carpinteria, CA, USA) and heated in a steamer at 97°C for 20 minutes. Anti-mouse F4/80 antibody (1∶200) was applied on tissue sections for 1 hour incubation at room temperature. Following washing, tissue sections were incubated with biotinylated anti-rat IgG (10 µg/ml, BA-4001, Vector Laboratories) for 30 minutes at room temperature. The antigen-antibody binding was detected by Elite kit (PK-6100, Vector Laboratories) and DAB (K3468, DAKO) system. Anti-Ki67 antibody (1∶200) was applied on tissue sections for overnight incubation at 4°C in a humidity chamber. Following washing, the antigen-antibody binding was detected with ImmPress AP anti-rabbit kit (MP-5401, Vector Laboratories) and Warp Red chromogen (WR806s, Biocare Medical, Concord, CA, USA). Tissue sections were briefly immersed in hematoxylin for counterstaining and were covered with cover glasses. By this protocol, Ki67 and F4/80 staining was indicated by red nuclear staining and brown cellular staining, respectively.

In another set of studies, slides were then incubated with rat anti-mouse CD11b, followed by goat anti-rat IgG and Streptavidin-peroxidase (Sigma/Aldrich, St. Louis, MO, USA). Specifically-bound antibodies were detected using a 3,3′-diaminobenzidine (DAB)-based technique (ImmPACT DAB, Vector Laboratories). The peroxidases and biotin were again blocked as before. Slides were then incubated with anti-mouse PCNA, followed by biotin-conjugated anti-rabbit IgG (Vector Laboratories) and Streptavidin-peroxidase (Sigma). Specific PCNA staining was then detected with the VIP Substrate Kit (Vector Laboratories), followed by methyl-green counter-staining (Vector Laboratories). By this protocol, PCNA and CD11b staining was indicated by purple nuclear staining and brown cellular staining, respectively.

### Flow Cytometry

Peripheral blood cells were collected via orbital draw at indicated times. Red blood cells were lysed with NH_4_Cl/KHCO_3_ and resuspended in FACS buffer (1× PBS, 2% calf serum, 5 mM EDTA, 0.1% sodium azide) and stained with the antibodies listed above.

Renal infiltrating cells were isolated as described previously [15]. In brief, mouse kidneys were minced and digested at 37°C for 30 min with gentle agitation with collagenase IV (2 mg/ml) in HBSS/1% (vol/vol) BSA (all from Sigma). Erythrocytes were lysed with NH_4_Cl/KHCO_3_ and the cell suspension was passed through a 40 µm cell strainer (BD Biosciences). Approximately one million cells from each kidney were stained with the antibodies listed previously. Flow cytometry was performed with a FLSRII (BD Biosciences) and analyzed with FlowJo software (Tree Star, Inc., Ashland, OR, USA).

### Statistical Analyses

Anderson-Darling tests were used to evaluate data normality (Minitab v. 16.2.4, State College, PA, USA). Comparisons between two groups of parametric data were made with two-sample t-testing. In the text, data are presented as means ± SEMs.

## Results

### TI Nephritis in Crry^−/−^C3^−/−^ Kidneys Transplanted into Complement-sufficient Mice

**Figure 1:**
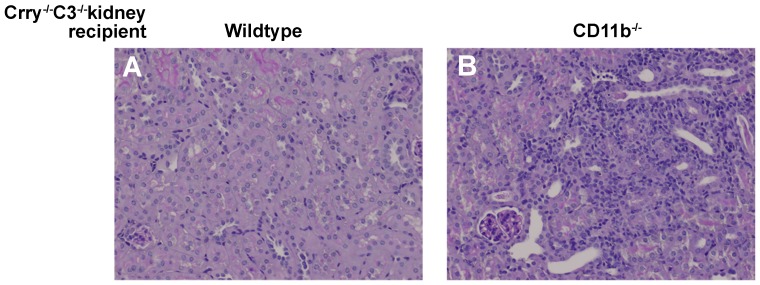
Light microscopic features of Crry^−/−^C3^−/−^ kidneys transplanted into wildtype and CD11b^−/−^ recipients. Shown are representative periodic acid-Schiff stained sections of Crry^−/−^C3^−/−^ kidneys transplanted 7 days earlier into wildtype and CD11b^−/−^ recipients. There was greater inflammation in CD11b^−/−^ recipients compared to wildtype recipients. TI nephritis scores ranged 2.0–2.5 and 3.0–4.0 in wildtype and CD11b^−/−^ recipients, respectively (n = 5 each).

**Figure 2:**
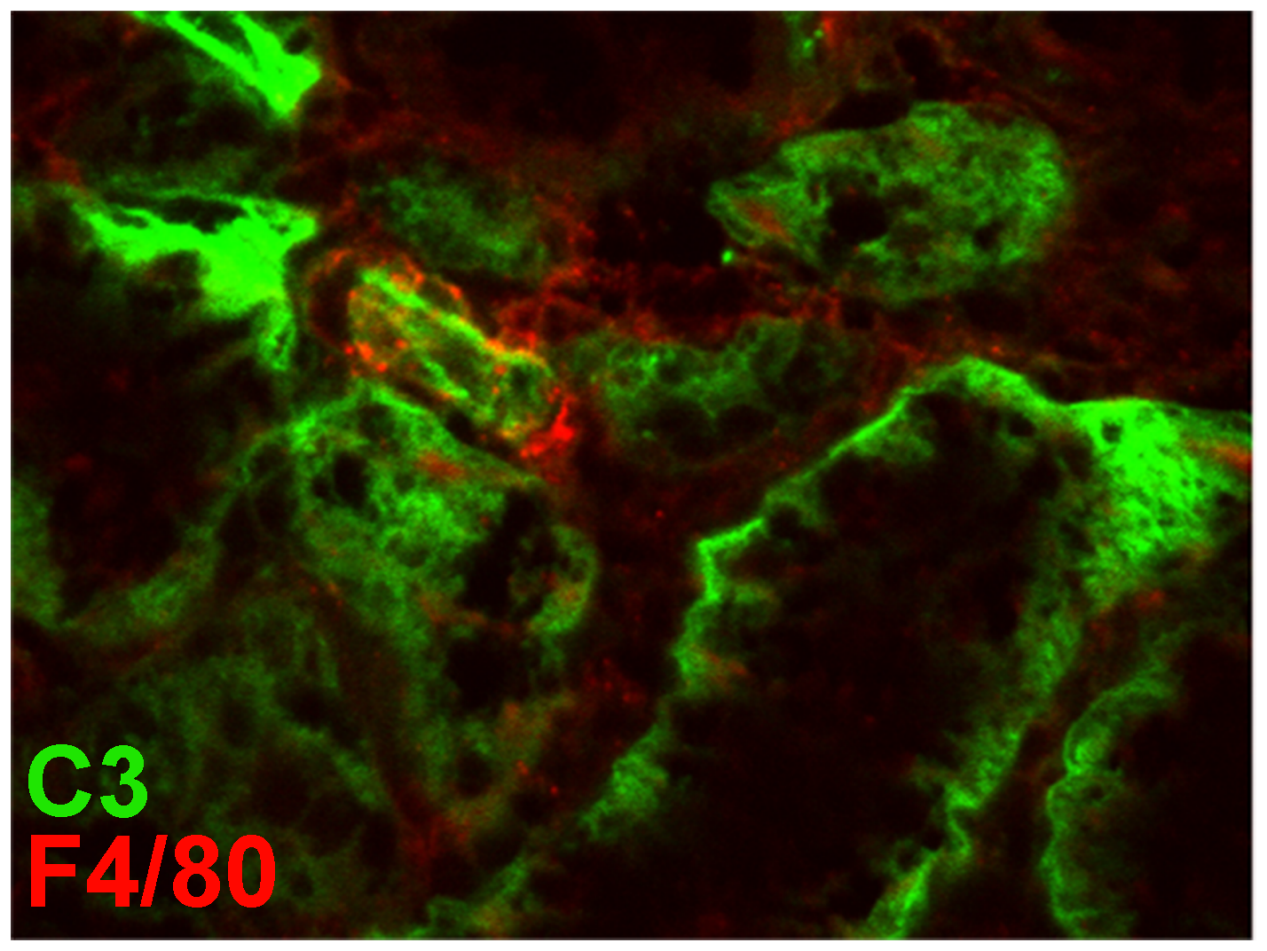
Dual immunofluorescence staining showing the association of F4/80^+^ cells with deposited C3. Shown is a representative immunofluorescence micrograph of a Crry^−/−^C3^−/−^ kidney 7 days after transplantation into a wildtype recipient. Considerable deposition of C3 (green) is evident in the basolateral aspects of tubules, along with closely approximated F4/80^+^ cellular processes (red).

Kidneys from Crry^−/−^C3^−/−^ mice transplanted into wildtype hosts developed TI nephritis (scores 2.0–2.5) (**Figure 1**). C3^−/−^ kidneys transplanted into wildtype mice and Crry^−/−^C3^−/−^ kidneys transplanted into Crry^−/−^C3^−/−^ mice did not develop TI nephritis [9]. That F4/80^+^ mononuclear cells were in close proximity to the basolateral C3 deposits (**Figure 2**), led us to suspect iC3b-CD11b interactions were relevant. Yet, surprisingly, Crry^−/−^C3^−/−^ kidneys in CD11b^−/−^ recipients had a greater degree of inflammation and TI nephritis (scores 3.0–4.0). As we have documented in this model, there was extensive C3 deposition along the basolateral aspects of tubules, which was no different between the two groups. Thus, TI nephritis was unexpectedly worsened when the transplant recipient lacked CD11b.

### Peripheral Blood Monocytes

**Figure 3:**
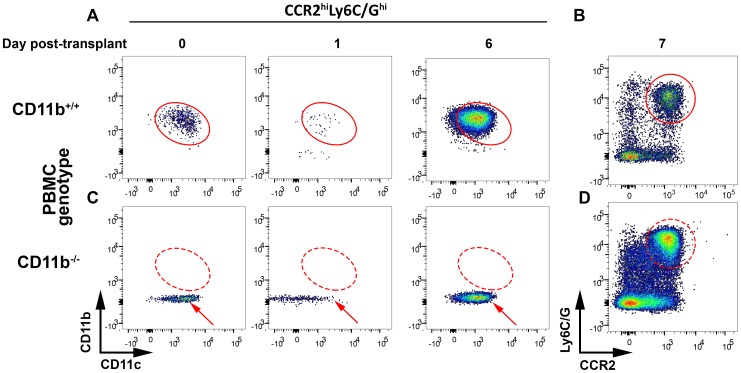
Fate of Gr1^+^/Ly6C^hi^ monocytes over time post-transplantation of Crry^−/−^C3^−/−^ kidneys into wildtype and CD11b^−/−^ recipients. Flow cytometry was performed on peripheral blood mononuclear cells obtained from wildtype (CD11b^+/+^, **A**, **B**) and CD11b^−/−^ animals (**C**, **D**) prior to and 1, 6 (**A**, **C**), or 7 days (**B**, **D**) after receiving Crry^−/−^C3^−/−^ kidney transplants. The CCR2^hi^Ly6C/G^hi^CD11b^+^CD11c^+^ cellular population prior to transplantation (day 0) is bounded by a red oval; its relative position is maintained in the subsequent panels for day 1 and 6 data. The positions of the analogous cellular populations in CD11b^+/+^ recipients are maintained in CD11b^−/−^ recipients and depicted with dashed lines. The red arrows point to CCR2^hi^Ly6C/G^hi^CD11b^−^CD11c^+^ cells. At each time point, blood was obtained from two animals in each group; shown are representative data from one of the two.

Circulating Gr1^+^/Ly6C^hi^ (inflammatory) monocytes [16], [17] also expressed CCR2 (**Figure 3B**, red circle), and both CD11b and CD11c (**Figure 3A**, red ovals). In wildtype mouse recipients of Crry^−/−^C3^−/−^ kidneys, there was an initial decline in these cells 1 day after transplant (**Figure 3A**), while they were expanded 7 days post-transplant (**Figure 3B**). Overall, CD11b^−/−^ transplant recipients had qualitatively similar findings, except for the complete absence of CD11b on inflammatory monocytes (**Figure 3C**, red arrows). The initial decline in inflammatory monocytes is likely attributable to their recruitment to the transplanted kidney (see below), while at later time points, they are recruited from bone marrow stores accounting for this monocytosis. Thus, local inflammation within transplanted kidneys generates a systemic inflammatory response, which does not appear to be affected to a significant extent in the absence of CD11b. The resident CD11b^+^ cells in the kidney do not appear in the peripheral circulation.

### Inflammatory Cells in Transplanted Crry^−/−^C3^−/−^ Kidneys

**Figure 4:**
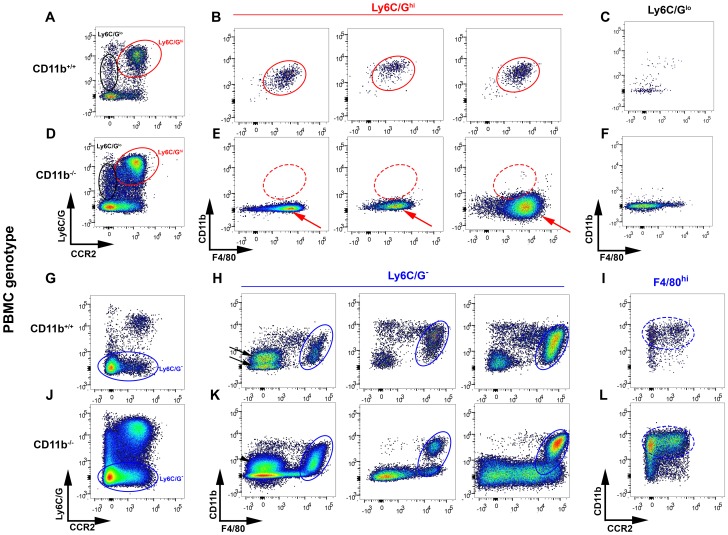
Cellular characterization of tubulointerstitial infiltrates in Crry^−/−^C3^−/−^ kidneys transplanted into wildtype and CD11b^−/−^ recipients. Cells isolated from Crry^−/−^C3^−/−^ kidneys 7 days following transplantation into wildtype (CD11b^+/+^, **A–C** and **G–I**) and CD11b^−/−^ animals (**D–F** and **J–L**) were analyzed by flow cytometry. In wildtype recipients, cells characteristic of M1 macrophages were CCR2^hi^Ly6C/G^hi^ (**A**, red oval) and F4/80^+^CD11b^+^ (**B**, red ovals). There were few CCR2^−^Ly6C/G^lo^ cells (**A**, black oval); these were also stained for CD11b and CCR2 (**C**). Ly6C/G^−^ cells included F4/80^hi^CD11b^+^ cells (**G** and **H**, blue ovals) which were analyzed for CCR2 and CD11c (**F**). There were Ly6C/G^−^F4/80^−^ cells (**G**) that were either CD11b^lo^ or CD11b^−^ (**H**, black arrows). The positions of the analogous cellular populations in CD11b^+/+^ recipients are shown in CD11b^−/−^ recipients and depicted with solid lines where their staining characteristics are comparable, and dashed lines when they are dissimilar from wildtype recipients. The red arrows point to CCR2^hi^Ly6C/G^hi^CD11b^−^CD11c^+^ cells (**E**). Data are from six separate Crry^−/−^C3^−/−^ kidneys, three each in wildtype and CD11b^−/−^ recipients.

Crry^−/−^C3^−/−^ kidneys in wildtype recipients had an inflammatory infiltrate which expressed high levels of Ly6C/G and CCR2 (**Figure 4A**, red oval), as well as surface F4/80 and CD11b (**Figure 4B**, red ovals). These characteristics indicate these are inflammatory (M1) macrophages, which likely originated from Ly6C/G^hi^CCR2^hi^ inflammatory monocytes shown in **Figure 3B** [18]. There were few Ly6C/G^lo^CCR2^−^CD11b^+^ neutrophils (**Figure 4A**, black oval, and **Figure 4C**), consistent with limited neutrophil inflammation in this model [10].

In contrast to these infiltrating cells, those cells that did not express Ly6C/G (**Figure 4G**, blue oval) were largely or exclusively mononuclear phagocyte (MPC) cells intrinsic to the kidney (discussed further below). These included cells that highly expressed F4/80 (**Figure 4H**, blue ovals), along with CD11b and CD11c, and variable amounts of CCR2 (**Figure 4I**). There were two Ly6C/G^−^F4/80^−^ cell populations, differing by the presence or absence of CD11b (**Figure 4H**, black arrows). The CD11b^lo^ cells included a population of CD11c^hi^ cells (data not shown).

Crry^−/−^C3^−/−^ kidneys in CD11b^−/−^ recipients were also infiltrated with Ly6C/G^hi^CCR2^hi^ inflammatory macrophages (**Figure 4D**, red oval); these were present in significantly greater numbers compared to wildtype recipients (19.4±4.3% vs 5.3±1.3%, P = 0.05). Consistent with their being recruited from inflammatory monocytes of the host animal, these Ly6C/G^hi^CCR2^hi^ cells lacked expression of CD11b (**Figure 4E**, red arrows). There were also Ly6C/G^lo^CCR2^−^ neutrophils (**Figure 4D**, black oval), which lacked CD11b as expected (**Figure 4F**), given their origin from the CD11b^−/−^ recipient.

There were Ly6C/G^−^F4/80^hi^CD11b^+^ and Ly6C/G^−^F4/80^−^CD11b^lo^ cells in Crry^−/−^C3^−/−^ kidneys in CD11b^−/−^ recipients (**Figure 4K**, blue ovals and black arrowhead, respectively) as there were in wildtype recipients.

The CD11b^+^ cells present in Crry^−/−^C3^−/−^ mouse kidneys in CD11b^−/−^ recipients must have originated from the transplanted kidney. In particular, the Ly6C/G^−^F4/80^hi^CD11b^+^ population (**Figure 4L**, dashed blue oval) was markedly expanded relative to baseline and Crry^−/−^C3^−/−^ kidneys in wildtype recipients (**Figure 4I**, dashed blue oval). This suggests these cells had proliferated *in situ*.

**Figure 5:**
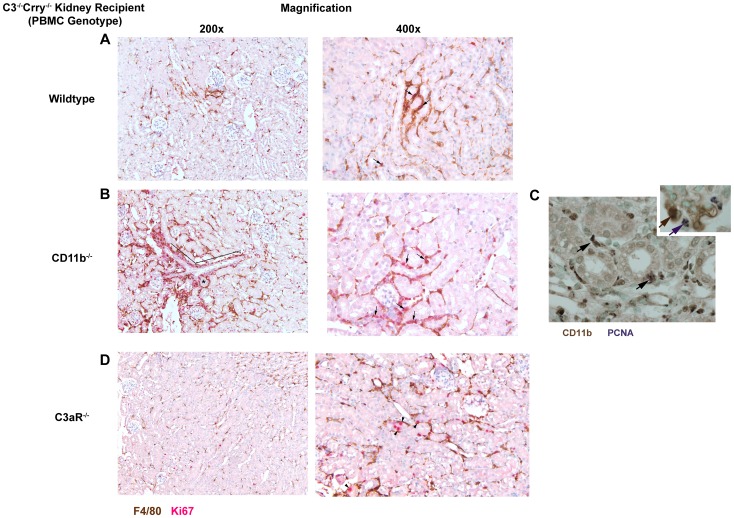
Dual immunohistochemical staining showing proliferation of F4/80^+^ and CD11b^+^ cells in Crry^−/−^C3^−/−^ kidneys transplanted into CD11b^−/−^ recipients. Shown is representative immunohistochemistry in Crry^−/−^C3^−/−^ kidneys transplanted 7 days earlier into wildtype (**A**), CD11b^−/−^ (**B**, **C**) and C3aR^−/−^ recipients (**D**). There was greater inflammation in CD11b^−/−^ recipients compared to wildtype and C3aR^−/−^ recipients. TI F4/80 cellular staining is brown and Ki67 is red (**A**, **B**, **D**); CD11b cellular staining is brown and PCNA nuclear staining is purple (**C**). The arrows depict cells that have both staining products. Arrowheads in **D** depict Ki67 staining in tubular cells. **Inset to C**, Glomerulus from murine lupus nephritis stained for CD11b and PCNA showing distinct reaction products. In each group, separate Crry^−/−^C3^−/−^ kidneys are shown magnified 200× and 400×.

To further evaluate whether these F4/80^hi^ cells were entering the cell cycle and proliferating, we double stained Crry^−/−^C3^−/−^ kidneys for F4/80 and Ki67. Crry^−/−^C3^−/−^ kidneys in wildtype hosts had peritubular collections of F4/80^+^ cells, some of which co-expressed Ki67 (**Figure 5A**, black arrows). As expected based upon histologic and flow cytometric data, Crry^−/−^C3^−/−^ mouse kidneys in CD11b^−/−^ recipients had considerably more F4/80^+^ cells in perivascular (**Figure 5B**, black line) and peritubular inflammation (**Figure 5B**, asterisk). Considerable numbers of these also expressed Ki67 (**Figure 5B**, black arrows). To confirm these cells originated from the kidney, we also stained for CD11b and PCNA. This revealed CD11b^+^ cells also stained positively for PCNA (**Figure 5C**, arrows).

To compare these results with our past studies showing the C3aR-dependence of the inflammation [10], we examined Crry^−/−^C3^−/−^ mouse kidneys in C3aR^−/−^ recipients for F4/80 and Ki67. As expected, there were F4/80^+^ cells throughout the TI as in normal kidneys but there was not significant inflammation (**Figure 5C**). Interestingly, there were cells expressing Ki67, which were in tubules (**Figure 5C**, black arrowheads), suggesting an ongoing repair process.

## Discussion

In these studies we utilized a model of TI nephritis in which acute C3 activation in Crry^−/−^C3^−/−^ kidneys generated C3aR-dependent inflammation [9], [10]. Here we have further characterized the TI cellular infiltrates. The Ly6C/G^hi^CCR2^hi^F4/80^+^CD11b^+^ cells are consistent with inflammatory M1 macrophages. Their accumulation in the kidney can be attributed to their recruitment from circulating inflammatory Gr1^+^/Ly6C^hi^ monocytes. This cellular pool was in turn considerably expanded by “emergency myelopoeisis” [18]. Thus, when the recipient animal was CD11b-deficient, the Ly6C/G^hi^CCR2^hi^F4/80^+^ inflammatory macrophages completely lacked CD11b.

Among the Ly6C/G^−^ cells were F4/80^hi^CD11b^+^CD11c^+^ cells. Since these correspond well to the MPC3 population recently described by Nelson, Duffield et al [19], we will follow their nomenclature. These MPC3 cells do have several characteristics of dendritic cells, including high CX_3_CR1 and MHCII expression, phenotypic features of dendritic processes [20], [21], and ability to serve as professional antigen presenting cells [22], [23]. Yet, their high F4/80 expression is atypical for dendritic cells, as is also true for variable CCR2 expression, including some with high expression indistinguishable from inflammatory macrophages. On average, inflammatory macrophages had greater CD11b and lesser CD11c surface staining than MPC3 cells, but there was considerable overlap in the aggregate populations. Overall, as noted recently by a panel of experts [24], renal MPCs have considerable overlap of macrophage and dendritic cell properties, making conventional binary naming systems inadequate.

Interestingly, there were two Ly6C/G^−^F4/80^−^ cell populations (i.e., depicted by the arrows in **Figure 4H**), which were distinguished by CD11b staining into CD11b^lo^ and CD11b^−^ cells. These are consistent with MPC4 and 5 subpopulations in the previously described study [19]. The MPC4 cells had considerable antigen-presenting potential, and were potentially CD103^+^ classical dendritic cells [19]. While we cannot comment on function, that these cells were expanded in Crry^−/−^C3^−/−^kidneys in CD11b^−/−^ hosts (c.f., arrowhead, **Figure 4K**), establishes their origin from the kidney itself.

These studies are not intended to model allotransplantation, as donor and recipient mice were genetically identical (but for the targeted genes). Yet, like with allotransplantation, the lymphatics are severed in the donor kidney. There were not any CD11b^+^ cells within the peripheral blood mononuclear cell pool in CD11b^−/−^ recipients of Crry^−/−^C3^−/−^ mouse kidneys. Thus, there was not emigration of any CD11b^+^ mononuclear cell population from the CD11b-sufficient transplanted kidney.

In normal kidneys, C3 (and C5b-9) staining is evident along the basement membranes of Bowman's capsule and renal tubules [25]. This is attributable to alternative pathway activation, and is enhanced in warm ischemic-reperfusion injury [5], [26]. A comparable sequence of events occurs in Crry/C3-deficient kidneys placed in a C3-sufficient environment, in which there is acute complement activation [[9] and unpublished data]. It is likely these pathological events mirror those destined to occur in acute infections, with a rapid response by inflammatory monocytes to generate inflammatory macrophages in tissue. These cells have direct effects on infectious microorganisms, and facilitate adaptive immune responses by lymphocytes. Once the infection is cleared, there is resolution of inflammation and return of the organ to normal.

Complement activation in the renal TI from excessive activation and/or abnormal regulation appears relevant in a number of disease states. Ammonia can hydrolyze C3 to initiate the alternative pathway. Given heightened ammoniagenesis, complement activation may have a role in progression of renal diseases of diverse origins [26], a theory supported by contemporary clinical data [27], [28]. Alloantibody-mediated complement activation appears etiologic in humoral renal allograft rejection [29]. Inherited and acquired abnormalities of membrane cofactor protein, a complement regulator present in the renal TI [2], [30] can underlie atypical hemolytic uremic syndrome and acute kidney injury, respectively [8]. Given their similar distributions and shared functions, our findings with Crry in the kidney are relevant to these human disease states in which there is abnormal complement regulation by membrane cofactor protein [31].

While the role for β2 integrins in cellular adhesion is well established, there is growing appreciation for their involvement in cellular signaling. Anaphylatoxin and chemokine receptor activation, including C3aR, recruit G_i_α leading to “inside-out” activation of CD11b and increased avidity for ligand [32], [33] such as iC3b [34]. When CD11b binds its ligand it can generate “outside-in” tyrosine kinase signals mediated by immunoreceptor tyrosine-based activation motif (ITAM) proteins, DAP12 and FcγR; mononuclear phagocytic cells primarily rely on DAP12 [35], [36]. Of considerable interest is the anti-inflammatory nature of these signals through CD11b. For example, CD11b can limit pro-inflammatory signals through Toll-like receptor (TLR) 4 activation by E3 ubiquitin-protein ligase CBL-B-mediated removal of activated proteins [37], [38], [39].

The expanded Ly6C/G^−^F4/80^hi^CD11b^+^CD11c^+^ MPC3 cellular population in CD11b-deficient recipients could only have come from the transplanted kidney. That this was due to intrinsic proliferation was confirmed by Ki67 and PCNA staining. These appeared to retain the original phenotypic features of MPC3 cells in the normal uninflamed kidney. Traditional views were tissue-resident macrophages had limited self-renewal properties and the expansion of macrophages in inflammation was due to recruitment from blood monocytes [40]. However, it is now clear macrophages in a variety of tissue sites have proliferative capacity [41], [42], [43], [44], [45]. Similarly to the MPC3 cells described here, these typically are F4/80^hi^ and are distinct from monocyte-derived macrophages.

## Conclusions

In conclusion, in our study we used a model of TI nephritis, in which acute C3 activation leads to C3a generation and deposition of C3b in the TI. When CD11b is absent on monocytes, TI nephritis was worsened. This was due both to greater inflammation with Gr1^+^/Ly6C^hi^ inflammatory monocyte-derived inflammatory M1 macrophages and expansion of the Ly6C/G^−^F4/80^hi^ intrinsic renal MPC3 cellular pool.

Thus, CD11b^+^ monocytes limit their own infiltration into the kidney and prevent proliferation of endogenous CD11b^+^ MPC cells. This suggests a role for outside-in iC3b-CD11b signals in limiting intrinsic organ inflammation.
